# Effects of Whole-Body Vibration Therapy in Patients with Fibromyalgia: A Systematic Literature Review

**DOI:** 10.1155/2015/719082

**Published:** 2015-08-17

**Authors:** Daniel Collado-Mateo, Jose C. Adsuar, Pedro R. Olivares, Borja del Pozo-Cruz, Jose A. Parraca, Jesus del Pozo-Cruz, Narcis Gusi

**Affiliations:** ^1^Faculty of Sport Sciences, University of Extremadura, 10003 Caceres, Spain; ^2^Universidad Autónoma de Chile, 3460000 Talca, Chile; ^3^Department of Sport and Exercise Science, The University of Auckland, Auckland 1142, New Zealand; ^4^Faculty of Sport and Physical Education, University of Évora, 7005-399 Évora, Portugal; ^5^Department of Physical Education and Sport, University of Seville, 41013 Seville, Spain

## Abstract

*Objective*. To review the literature on the effects of whole-body vibration therapy in patients with fibromyalgia. *Design*. Systematic literature review. *Patients*. Patients with fibromyalgia. *Methods*. An electronic search of the literature in four medical databases was performed to identify studies on whole-body vibration therapy that were published up to the 15th of January 2015. *Results*. Eight articles satisfied the inclusion and exclusion criteria and were analysed. According to the Dutch CBO guidelines, all selected trials had a B level of evidence. The main outcomes that were measured were balance, fatigue, disability index, health-related quality of life, and pain. Whole-body vibration appeared to improve the outcomes, especially balance and disability index. *Conclusion*. Whole-body vibration could be an adequate treatment for fibromyalgia as a main therapy or added to a physical exercise programme as it could improve balance, disability index, health-related quality of life, fatigue, and pain. However, this conclusion must be treated with caution because the paucity of trials and the marked differences between existing trials in terms of protocol, intervention, and measurement tools hampered the comparison of the trials.

## 1. Introduction

Fibromyalgia (FM) is a chronic disorder of unknown aetiology. It is characterised by widespread noninflammatory pain and tenderness that persists for at least 3 months and by an acute response in at least 11 of 18 specified tender points when these points are digitally palpated with a pressure of 4 kg/cm^2^ [1]. FM is associated with several symptoms, including fatigue, disrupted sleep, impaired cognition, poor physical fitness, headaches, arthritis, muscle spasm, tingling, and balance problems [2, 3]. These symptoms reduce the health-related quality of life (HRQoL) of people with FM [4].

In European populations (Spain, Portugal, France, Germany, and Italy), the estimated overall prevalence of FM is between 2.9% and 4.7% [5]. Consequently, this disorder imposes a significant economic burden due to healthcare costs and the inability of the patients to work [6]. In fact, FM accounts for 4–20% of all new patient contacts in primary care settings [7].

Several therapies have been used to treat the symptoms associated with FM, including pharmacological and nonpharmacological therapies. There is strong evidence showing that both pharmacological and nonpharmacological approaches can be effective [8]. Nonpharmacological therapies include physical therapies such as yoga, tai chi, walking, and whole-body vibration (WBV) [9]. WBV is a physical therapy that was shown to improve muscle strength [10], body balance [11], gait mobility [12], cardiorespiratory fitness [13], bone-density [14], and pain [15] in healthy and various clinical populations.

WBV can be delivered by two types of exercise platform. One is a sinusoidal vibration device that induces reciprocal vertical displacements on the left and right sides of a fulcrum and generates higher lateral acceleration than vertical acceleration. The second is a vertical vibration device that induces up-and-down oscillations over a vertical axis and produces high strain in the vertical axis [16]. The intensity of vibration is determined by three parameters [17], namely, amplitude, frequency, and oscillation acceleration.

Some studies suggested that WBV therapy may improve balance, pain, and fatigue. The mechanisms behind these effects are not clear but may relate to the following.

1. WBV elevates heart rate and oxygen uptake, which could translate to improved cardiorespiratory fitness over the long term [18].
2. WBV may influence the neuromuscular system and improve reflex responses, especially in patients who have altered reflex generation. This may be related to the ability of vibration to (a) stimulate subcutaneous proprioceptors, (b) activate muscle spindles, thereby causing muscle contraction, and (c) stimulate Golgi tendon organs, thereby improving tonic and antagonist vibration reflexes [19]. WBV may also promote adaptation of human cutaneous sensors in the sole of the foot [20].
3. WBV may reduce the perception of pain because vibration affects the afferent discharge of fast adapting mechanoreceptors and muscle spindles [21].

Potential harmful effects of vibration exposure have been found in industrial workers, and vibration is recognised as an industrial hazard. Prolonged exposures can induce vascular and neurological injuries, and legal limits have been set in numerous countries [22]. Therefore, any training protocol has to maximize the potential benefits while reducing the potential adverse side effects. To our knowledge, WBV-related adverse effects have not been reported in any studies that focused on FM.

To our knowledge, there is one review that examined the effect of WBV therapy on FM-associated symptoms [23]. However, this review only examined three articles, all of which were published between 2008 and 2010. Another five articles on WBV therapy in FM have been published ever since. The aim of the present review was to provide an up-to-date analysis of the research on the effect of WBV on FM-associated symptoms, including poor balance, fatigue, pain, and poor HRQoL. The ultimate objective was to provide future directions in clinical practice.

## 2. Materials and Methods

Preferred Reporting Items for Systematic Reviews and Meta-Analyses (PRISMA) methodology was employed to carry out this systematic review [24].

### 2.1. Electronic Database Searches and Article Selection Strategy

To locate the articles reported in this systematic review, four well-known electronic databases were selected, and a list of terms and compounded terms was prepared. These activities were supervised by medical library science experts and experts in the field of WBV in FM. The databases were the Cochrane Library (2003–present), the Physiotherapy Evidence Database (PEDro; 2003–present), PubMed (1973–present), and TRIP (2002–present). The articles were located using the keywords “fibromyalgia” and “vibration” and the Boolean operator “AND”. Duplicate articles were manually removed by one of the authors.

**Figure 1:**
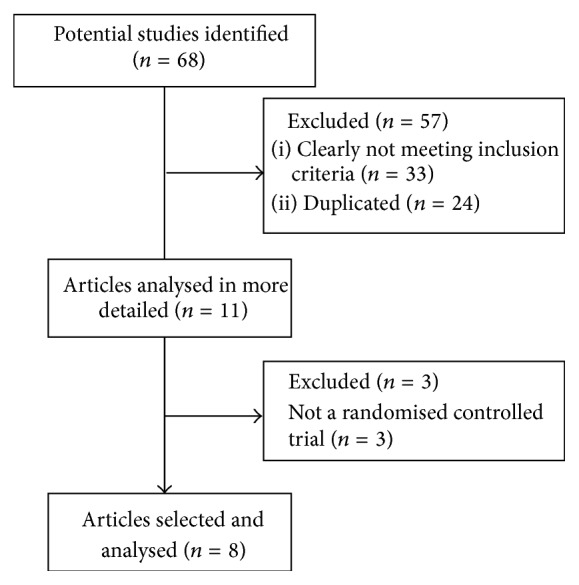
Flow chart delineating the complete systematic review process that was followed.

Figure 1 shows a flow chart delineating the complete systematic review process. The articles were indexed according to whether they met all of the following inclusion criteria: (a) the study focused on WBV therapy, (b) the study cohort only consisted of people with FM, (c) the study was a randomised controlled trial (RCT), (d) the whole publication was written in English, and (e) the article was an original clinical study. Studies were excluded if they met any of the following criteria: (a) the study examined the effects of exposure to vibration within industry or employment of labour and transport and (b) the study was only presented once as a summary at a conference, congress, or seminar. The articles were selected by two independent experts. Disagreements were resolved through group discussions until a mutual consensus was reached. The search was finalized on the 15th of January 2015, with no submission deadline being imposed.

### 2.2. Assessing the Risk of Bias

The PEDro scale was used to assess the risk of bias in the selected articles. This is a scale that rates the methodological quality of RCTs that evaluate physical therapist interventions. This scale was chosen because of its special design and capacity to provide a global overview of the external and internal validity of the studies [25]. Each article was graded by one of the authors, and this grading was supervised by another author with experience in this task.  Table 1 shows the consensus results for each article.

### 2.3. Determining the Level of Evidence

The level of evidence was determined using the guidelines of the Dutch Institute for Healthcare Improvement (CBO) [26].  Table 1 shows the results.

### 2.4. Data Extraction and the Main Measurements Examined

Data were extracted from the selected articles by one of the authors. This extraction was checked by another author. Any disagreement was discussed and ultimately resolved by a third author if the contact with the original author of the article could not be established.

For each selected article, the following data were extracted: (a) the sample and protocol characteristics, namely, the sample size, age, and activity of the control and WBV groups (Table 2) and (b) the vibration therapy details, namely, the type of device and its oscillation acceleration, frequency, and amplitude; the duration of the intervention; the number of WBV sessions; and the number of vibration series, the rest period, and the exposure duration in each series (Table 3).

## 3. Results

### 3.1. Article Selection

Figure 1 depicts the process that was followed in this systematic review. In total, 68 articles were found in the electronic search of the Cochrane Library (11 articles), PubMed (24 articles), Trip (25 articles), and PEDro (eight articles). After removing the duplicates, 44 references were reviewed. Of these, 33 were excluded because a review of their summaries revealed that the study clearly did not meet the inclusion criteria. The remaining 11 articles were then analysed in more depth to determine whether they satisfied the inclusion and exclusion criteria. This led to the exclusion of three articles because they were not RCTs. Finally, eight articles belonging to four different trials were included in our systematic review.

### 3.2. Risk of Bias

Table 1 shows the risk of bias of each of the four RCTs, as indicated by the PEDro scale score. All 8 articles were on RCTs because this was an inclusion criterion. The PEDro scale score ranged from 6 to 8 points (the maximum score was 10 points). The average (SD) score was 6.88 (0.83). The poorest scores were obtained for questions five (“there was blinding of all subjects”), six (“there was blinding of all therapists”; this reflects the fact that it is difficult to blind a WBV therapist), and nine (“intention-to-treat analysis was performed on all subjects who received the treatment or control condition as allocated”). Good scores were obtained for questions one (“eligibility criteria were specified”), two (“subjects were randomly allocated to groups or to a treatment order”), four (“the groups were similar at baseline”), eight (“measures of at least one key outcome were obtained from more than 85% of the subjects initially allocated to groups”), 10 (“the results of between-group statistical comparisons are reported for at least one key outcome”), and 11 (“the study provides both point measures and measures of variability for at least one key outcome”).

### 3.3. Level of Evidence

Table 1 also indicates the level of evidence in each study. All eight articles had a B level of evidence because; although every article reported the results of an RCT, none of these RCTs was double-blind.  Table 4 shows the level of conclusion according to the Dutch CBO guidelines (in the appendix). The score ranged from 2 to 3 because only four different RCTs were reviewed, all of which had a B level of evidence.

### 3.4. Study Characteristics

Tables 2, 3, and 5 summarize the study characteristics using the PICOS (Patients, Intervention, Control, Outcomes, and Study design) approach [24]. All four RCTs were performed with adult and elderly women with FM, and the sample size varied from 24 [27] to 46 [28] participants.

### 3.5. WBV Equipment

**Figure 2:**
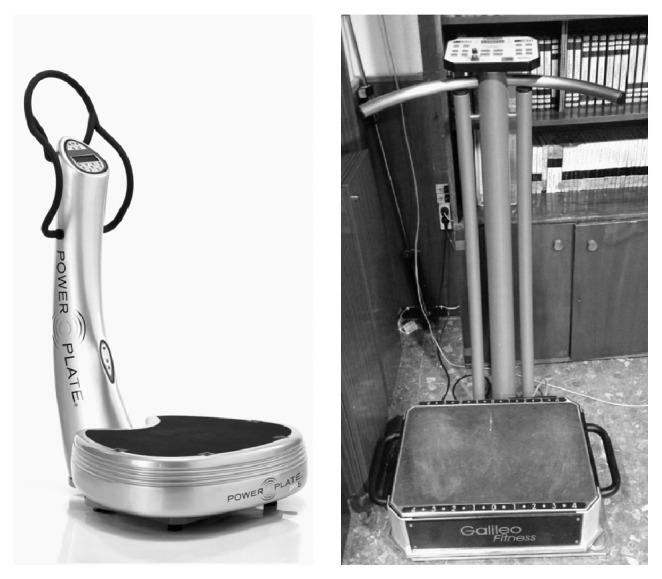
Power Plate and Galileo vibration platforms.

Two RCTs used the Power Plate vibratory platform [27–29], and the other two RCTs used the Galileo vibratory platform [30–34]. The Galileo platform produces a horizontal sine-wave vibration, whereas the Power Plate platform produces a vertical sine-wave vibration (Figure 2).

### 3.6. WBV Parameters

#### 3.6.1. Frequency and Amplitude

The four RCTs differed in terms of the amplitude and frequency of the vibration. The two RCTs that used the horizontal sine-wave vibration employed an amplitude of 2-3 mm and a frequency of 12.5–20 Hz, and the two RCTs that used the vertical sine-wave vibration employed an amplitude of 2–4 mm and a frequency of 30 Hz.

#### 3.6.2. Performance on the Platform

The postures used in the four RCTs also varied. In three RCTs, patients maintained a static posture on the platform during vibration [28, 30–34], whereas, in the fourth RCT, patients performed both static and dynamic tasks during vibration [27, 29]. In two RCTs, both feet were always on the platform during vibration [27, 29–32], and, in the other two RCTs, some series were performed with only one foot on the platform [28, 33, 34]. The knee angle varied between 45° and 130° in the two static-task RCTs and between 90° and 180° in the dynamic-and-static-task RCT.

#### 3.6.3. Description of Training

All four RCTs sought to analyse the long-term effects of WBV therapy. In two of the three RCTs, 6 weeks of WBV therapy were performed [27, 29, 33, 34]. The authors of the first RCT [27, 29] chose this duration because, although it would be inadequate for a traditional exercise program, it should be sufficient for the development of WBV-induced adaptations that would improve pain, fatigue, stiffness, and depression in patients with FM. The Gusi et al. RCT had the longest WBV therapy, which consisted of 36 sessions over 12 weeks [30–32]. The number of series ranged from three to ten, and each lasted between 30 s and 60 s with a rest interval of 45–180 s (Table 3).

### 3.7. Key Measurements and Effects

The measurements with the highest level of conclusion (Table 4) were balance, HRQoL, fatigue, and disability assessed using the fibromyalgia impact questionnaire (FIQ) [35]. The level of conclusion for pain was lower than that for the other measures, but because pain is a main symptom of FM, it was analysed independently.

#### 3.7.1. Balance

Three of the RCTs [28, 30–34] evaluated the effects of WBV therapy on balance, specifically dynamic balance and static balance. One of these RCTs analysed static balance with both open and closed eyes. All three RCTs used a Biodex Balance System to measure balance. This device measures the tilt about each axis during dynamic conditions and calculates a mediolateral stability index, an anteroposterior stability index, and an overall stability index [36]. These indices are SDs that assess fluctuations around a zero point that is established prior to testing when the platform is stable (rather than around the group mean). A lower score indicates better balance.

One study showed that WBV significantly improved the dynamic balance of the WBV group compared to the control group [30]. In three studies, mediolateral and anteroposterior indices were both measured, but, in two studies, only the mediolateral stability index improved [28, 34], and, in the other study, only the anteroposterior stability index improved [32].

#### 3.7.2. Quality of Life

Two studies assessed HRQoL [31, 33] using the global score of the 36-Item Short Form Health Survey (SF-36, Medical Outcome Study) [33] or the 15D^©^ questionnaire [31]. Both questionnaires are not specifically developed for FM. The 15D^©^ questionnaire assesses 15 HRQoL dimensions, namely, moving, seeing, hearing, breathing, sleeping, eating, speech, eliminating, usual activities, mental function, discomfort and symptoms, depression, distress, vitality, and sexual activity [37]. The SF-36 is a well-known questionnaire that assesses limitations, bodily pain, vitality, mental health, and general health perception [38]. Significant improvement in SF-36 score [33] was reported, but not in the 15D^©^ [31].

#### 3.7.3. Fatigue

Three of the RCTs assessed the effect of WBV therapy on fatigue [28, 29, 33]. Several different measures of fatigue were used: the number of repetitions of half squat performed in 60 s; a fatigue index, expressed as the decline in the peak torque from the start of the half squat exercise (first five repetitions) to the end of the exercise (last five repetitions); and the 100 mm visual analogue scale contained in the FIQ.

In one study, WBV improved fatigue relative to both baseline and the control group [29]. In another study, WBV improved the number of repetitions relative to baseline [33]. In the third study, WBV did not have a significant effect on fatigue [28].

#### 3.7.4. Disability Index

The disability caused by FM was assessed in three RCTs using the Spanish version of the FIQ [39]. One study [31] used this questionnaire to evaluate HRQoL. However, the FIQ is an instrument that assesses the effect of FM symptoms on health status and the disability index and does not measure HRQoL [40].

In one RCT [30–32], the WBV-treated patients exhibited a significant improvement in FIQ score relative to the untreated control patients. In the other two RCTs [27, 29, 33, 34], patients undergoing WBV during traditional exercises showed significant improvements in FIQ score relative to baseline. However, there were no statistically significant differences between the traditional exercise − only group and the exercise + vibration group.

#### 3.7.5. Pain

Pain is the most important symptom in FM, but it was assessed specifically in only one study, which reported an improvement in pain compared to both baseline and the control groups [29]. However, pain is part of the FIQ and 15D^©^ questionnaires that were used to evaluate HRQoL in two of the other RCTs [31, 33].

## 4. Discussion

The four RCTs revealed that WBV therapy may improve several symptoms of FM, namely, disability, pain, poor HRQoL, poor balance, and fatigue.

The duration of the treatment could be extremely relevant when assessing the effects of WBV on disability caused by FM. Two RCTs only involved 6 weeks of complementary vibration therapy, and one RCT involved 12 weeks of vibration therapy. This latter RCT reported a significant improvement in FIQ score relative to the control group, whereas the other two RCTs only reported within-group improvements. It may be that the effect of WBV therapy on FIQ score can only be observed with longer treatments.

With regard to pain, the single study examining this outcome used the 100 mm VAS to show that WBV therapy significantly improved pain compared to both baseline and the control groups [29]. However, the level of conclusion for this measure was 3, which is low. Pain is also a subscale of the FIQ. Three articles assessed the effects of vibration on total FIQ score, but they did not report the changes in this dimension. In patients with chronic back pain, evidence suggests that vibration could alleviate pain [41, 42]. The mechanism by which WBV could reduce pain perception was discussed widely in a previous review [21]. However, it is possible that the mechanisms that lead to a reduction of pain in diseases characterised by local pain do not work in FM patients, because FM is characterised by widespread pain, and the cause of pain is likely to be different. Because pain is one of the main symptoms of FM, additional studies that assess the effects of WBV on pain in FM are needed.

There were large differences in treatment effects on balance. These discrepancies may reflect differences between the WBV protocols. Effects of the two vibratory platforms (Galileo and Power Plate) on balance were compared by Sañudo et al. [28, 33, 34], who reported that the mediolateral stability index was improved by both the Galileo platform and the Power Plate platform. However, in the study of Adsuar et al., which used the Galileo platform, only the anteroposterior stability index improved [32]. Given that mediolateral sway is more correlated with fall risk [43], the protocol of Sañudo et al. may be better at preventing falls.

With regard to fatigue, Sañudo et al. compared the two vibratory platforms with the same protocol. They found that only the Galileo platform induced a significant improvement in the number of repetitions of a half squat exercise performed in 60 s. However, it cannot be concluded that these improvements were due to an improvement in cardiorespiratory fitness. Devices that assess oxygen consumption in a more objective way must be used to evaluate this measure.

Three RCTs yielded five articles [27–29, 33, 34] that showed that when WBV therapy complemented a physical exercise programme, there were significant improvements in muscle performance (increased strength and decreased fatigue) or balance that were larger and more significant than the improvements obtained by standard exercise (Table 5). Thus, adding WBV can enhance the benefits of a physical exercise programme. Given that WBV only requires a few minutes to be delivered, it could be a good complement to usual physical exercise protocols.

The differences between studies in the effect of WBV on balance [28, 32, 34], fatigue [28, 29, 33], and FIQ score [29, 31, 33] could be attributed to differences in the baseline scores or differences in the vibration protocols in terms of the type of vibration, the duration of treatment, and the rest intervals. The disparities could also reflect differences in the instrument that was used in the evaluation. In support of this, the baseline FIQ scores in the Sañudo et al. and Alentorn-Geli et al. studies differ by almost 28%; thus, it can be expected that these studies will differ in the degree of improvement that is observed. Other variables should be considered to better explain these differences, including the weather [44], patient weight [45], and patient age, which may be a surrogate marker of the effect of menopause on women with FM [46].

The four RCTs differed markedly in terms of important characteristics, namely, the type of vibration (vertical or horizontal sine wave), the type of therapy (vibration or exercise + vibration), and the vibration protocol (frequency, amplitude, time series, rest interval, and duration). Therefore, additional studies that assess the effect of WBV in different settings are needed. These studies should compare (a) different protocols with the same device and the same type of therapy, (b) different devices with a similar protocol and the same type of therapy, and (c) different types of therapies with the same device and the same protocol. These studies will identify the optimal characteristics of vibration therapy that are needed to improve functional capacity, HRQoL, balance, and other key symptoms of FM.

To our knowledge, the acute effects of WBV on FM symptoms have not been assessed by any study. However, a study on patients with low back pain showed that a single WBV session induced statistically significant within-group changes in lumbopelvic pain perception [47]. Another study on patients with low back pain reported that WBV therapy induced a greater reduction in pain after 12 months than after 6 months [42]. This suggests that the duration of WBV in the RCTs analysed in this study (6–12 weeks) was too short to significantly reduce pain. Therefore, additional trials that assess the effects of both short- and long-term (6–12 months) WBV therapy on FM-associated pain are needed. Studies that examine the effects of long-term WBV therapy on balance are also needed because several studies showed that long WBV treatments (6–12 months) significantly improved postural control and static and dynamic balance in populations that share some of the characteristics of patients with FM (i.e., postmenopausal or elderly women) [48–50].

The present systematic review only identified a limited number of studies on the effects of WBV therapy on FM. This reflects the fact that the RCTs on this issue only started very recently; the first completed study was published in 2008. The small number of trials together with their wide variation in terms of PICOS (Participants, Intervention, Control, Outcome Measurements, and Study design) hampers meta-analysis. This explains why the CBO guidelines [26] indicated that the level of conclusion regarding the effect of WBV therapy on FM was 3.

There are several limitations that should be considered. First, standardised criteria to assess the level of evidence are needed. Authors of systematic reviews often use different criteria [51] that depend on the methodological quality (i.e., RCT versus low-quality trials) of the analysed studies. The scales of measurement may vary across the criteria, and the best method for assessing the risk of bias is not clear [51]. However, only RCTs were considered in the current systematic review. Second, some bias may have been introduced because the search strategy omitted articles in languages other than English. Significant results are easier to publish than nonsignificant results and, consequently, the latter are more likely to appear in national journals that are written in a native language [52]. WBV dose-response analysis was not included because of the variability among devices and protocols, and the few available references in FM patients.

As emphasised in the Introduction, FM entails a huge cost to governments. Therefore, studies on the cost-effectiveness and utility of WBV as a therapy in a condition that is as prevalent and widespread as FM are needed.

## 5. Conclusions

WBV may be an adequate treatment for FM as a main therapy or when added to a physical exercise programme as it could improve the balance, disability index, quality of life, fatigue, and pain of patients with FM. However, the small number of RCTs on WBV in FM and their wide variation in terms of vibration protocol, intervention, and measurements hampered our comparison of these trials. Additional studies that definitively clarify the effects of WBV therapy on FM are needed.

## Figures and Tables

**Table 1:** Risk of bias and level of evidence.

Clinical trial	Reference	Response to each item on the PEDro scale												Level of evidence
		1	2	3	4	5	6	7	8	9	10	11	Total score	
Alentorn-Geli et al.	[29]	y	y	n	y	y	n	y	y	n	y	Y	7	B
	[27]	y	y	n	y	y	n	n	y	n	y	Y	6	B

Gusi et al.	[30]	y	y	y	y	n	n	y	y	y	y	Y	8	B
	[31]	y	y	y	y	n	n	y	y	y	y	Y	8	B
	[32]	y	y	y	y	n	n	y	y	n	y	Y	7	B

Sañudo et al.	[33]	y	y	y	y	n	n	n	y	n	y	Y	6	B
	[34]	y	y	y	y	n	n	n	y	n	y	Y	6	B

Sañudo et al.	[28]	y	y	y	y	n	n	n	y	y	y	Y	7	B

n: criterion not fulfilled; y: criterion fulfilled; 1: eligibility criteria were specified; 2: subjects were randomly allocated to groups or to a treatment order; 3: allocation was concealed; 4: the groups were similar at baseline; 5: there was blinding of all subjects; 6: there was blinding of all therapists; 7: there was blinding of all assessors; 8: measures of at least one key outcome were obtained from more than 85% of the subjects who were initially allocated to groups; 9: intention-to-treat analysis was performed on all subjects who received the treatment or control condition as allocated; 10: the results of between-group statistical comparisons are reported for at least one key outcome; 11: the study provides both point measures and measures of variability for at least one key outcome; total score: each satisfied item (except the first) contributes 1 point to the total score, yielding a PEDro scale score that can range from 0 to 10. B: the level of evidence was B (randomised control trials that lacked double-blinding) (see the Appendix).

**Table 2:** Sample characteristics and protocol.

Clinical trial	Sample characteristics			Protocol		
	Reference	Total sample size (*n*)	Age of whole cohort (mean ± SD) (years)	Treatment of the control group(s)	Treatment of the vibration group	WBV protocol
Alentorn-Geli et al.	[27] [29]	24^*^ 33^*^	54.95 ± 2.03 55.97 ± 1.55	Static and dynamic tasks on a vibratory platform without vibratory stimulus EG: traditional physical exercise plus static and dynamic tasks on a vibratory platform without vibratory stimulus CG: no exercise programme	Static and dynamic tasks with a WBV protocol Traditional exercise, followed by static and dynamic tasks with a WBV protocol	Static and dynamic tasks on a vibratory platform with vibratory stimulus: (a) Static squat at 100° of knee flexion (b) Dynamic squat between 90° and 130° of knee flexion (c) Maintenance of ankle plantar flexion with legs in extension (d) Flexion-extension of the right leg between 100° and 130° of knee flexion (e) Flexion-extension of the left leg between 100° and 130° of knee flexion (f) Squat at 100° of knee flexion, shifting the body weight from one leg to the other

Gusi et al.	[31] [30] [32]	36^*^ 41^**^ 36^*^ 41^**^ 36^*^	41–65 41–65 41–65	None	WBV protocol	The stance of the participants on the platform alternated between the following: (a) feet placed perpendicular to the midline axis of the platform, with the right foot positioned slightly ahead of the left foot. The toes of the right foot and the heel of the left foot were then lifted 4 mm above the surface of the platform. The knees were bent and maintained at a 45° knee angle. (b) Feet were placed perpendicular to the midline axis of the platform, with the left foot positioned slightly ahead of the right foot. The toes of the left foot and the heel of the right foot were lifted 4 mm above the surface of the platform. The knees were bent and maintained at a 45° knee angle

Sañudo et al.	[34] [33]	26^*^ 26^*^	59 ± 7.9 59 ± 7.9	Physical exercise	Physical exercise plus WBV protocol	Three sets of 45 s performed with both feet in contact with the platform and four sets of 30 s with only one foot in contact with the platform (15 s per foot). In each set, the participants stood with both knees in 120° isometric knee flexion

Sañudo et al.	[28]	46^**^	57.15 ± 6.8	Physical exercise	Physical exercise plus WBV protocol	Six sets of 30 s performed with both feet on the platform and four sets of 60 s with only one foot in contact with the platform (30 s per foot) In each set, the participants stood with both knees in 120° isometric knee flexion

CG: control group; EG: exercise group; ∗*n* for treatment effects analysis; ∗∗*n* for intention-to-treat analysis.

**Table 3:** WBV therapy and activity.

Authors	Freq. (Hz)	Amp. (mm)	Vibration device	Type of vibration	Duration (weeks)	Number of sessions	Number of series	Time series (s)	Rest between series (s)
Alentorn-Geli et al. [27, 29]	30	2	Power Plate	Vertical sine wave	6	12	Three in the first two sessions Six in the last nine sessions	30	180

Gusi et al. [30–32]	12.5	3	Galileo	Horizontal sine wave	12	36	6	30 s in sessions 1–4 45 s in sessions 5–8 60 s in sessions 9–12	60

Sañudo et al. [33, 34]	20	3^*^ 2^**^	Galileo	Horizontal sine wave	6	18	3^*^ 4^**^	45^*^ 30^**^	120

Sañudo et al. [28]	30	4	Power Plate	Vertical sine wave	8	24	6^*^ 4^**^	30^*^ 60^**^	45

Freq.: frequency; Amp.: amplitude.

^*^With both feet in contact with the platform; ^**^With one foot in contact with the platform.

**Table 4:** Level of conclusion according to the Dutch CBO guidelines.

Outcome measure	Level of conclusion
Balance	2
Quality of life	2
Serum insulin-like growth factor-1	3
Strength	3
Fatigue	2
Pain	3
Depression	3
Stiffness	3
Disability index (fibromyalgia impact questionnaire)	2

CBO: Institute for Healthcare Improvement.

Level 2: one trial of level A2 or at least two independent trials of level B (see the Appendix); 3: one trial of level B or C (see the Appendix).

**Table 5:** Outcome measures.

Authors	Reference	Instrument	Outcome measure	CG baseline	CG after treatment	EG baseline	EG after treatment	Treatment effect	Reported effect
Alentorn-Geli et al.	[27]	ELISA	IGF-1	NR	NR	NR	NR	NR	=
	[29]	FIQ	Functional capacity	NR	NR	NR	NR	NR	Δ
		100 mm VAS	Pain	NR	NR	NR	NR	NR	Δ ↑
			Fatigue	NR	NR	NR	NR	NR	Δ ↑
			Stiffness	NR	NR	NR	NR	NR	↑
			Depression	NR	NR	NR	NR	NR	=

Gusi et al.	[31]	FIQ	Functional capacity	53.6 ± 12.3 55.27 ± 12.73^*^	57.5 ± 11.2 59.13 ± 11.71^*^	59.3 ± 9.8 56.89 ± 10.38^*^	56.7 ± 11.1 55.40 ± 11.41^*^	−6.42 −5.35^*^	↑ ↑
		15D questionnaire	Quality of life	0.65 ± 0.1	NR	0.63 ± 0.1	NR	NR	=
	[30]	Biodex Balance System	Dynamic balance	1.47 ± 0.55 1.40 ± 0.55^*^	1.51 1.43^*^	1.49 ± 0.67 1.59 ± 0.73^*^	0.85 1.02^*^	−0.69 −0.60^*^	↑ ↑
	[32]	Biodex Balance System	SB overall SI (°)	1.36 ± 0.50	1.40 ± 0.50	1.53 ± 0.56	0.88 ± 0.41	−0.65	↑
			SB anteroposterior SI (°)	0.80 ± 0.29	0.96 ± 0.47	1.05 ± 0.49	0.56 ± 0.31	−0.64	↑
			SB mediolateral SI (°)	0.94 ± 0.37	0.83 ± 0.26	0.88 ± 0.37	0.55 ± 0.22	−0.19	=

Sañudo et al.	[34]	T-Force Dynamic Measurement System	Knee extensor strength	207.4 ± 16.5	202.6 ± 13.6	208.2 ± 16.7	210.7 ± 18.1	7.3	=
		Biodex Balance System	OE overall SI	7.50 ± 3.07	6.47 ± 2.98	6.63 ± 3.23	5.5 ± 2.67	−0.1	=
			OE mean deflection	6.25 ± 2.93	5.63 ± 2.86	5.52 ± 2.87	4.66 ± 2.55	−0.2	=
			OE anteroposterior SI	5.42 ± 2.13	5.33 ± 2.68	4.97 ± 2.45	4.76 ± 2.81	−0.1	=
			OE anteroposterior mean deflection	2.93 ± 1.37	3.48 ± 2.64	2.90 ± 1.79	3.60 ± 2.81	0.2	=
			OE mediolateral SI	5.07 ± 2.57	3.35 ± 1.97	4.34 ± 2.32	2.49 ± 1.09	−0.1	↑
			OE mediolateral mean deflection	1.61 ± 1.18	1.23 ± 0.93	1.28 ± 1.04	1.17 ± 0.75	0.3	=
			CE overall SI	11.67 ± 2.41	11.37 ± 2.32	11.9 ± 2.16	10.7 ± 2.64	−0.9	=
			CE mean deflection	10.35 ± 2.25	9.90 ± 2.30	10.51 ± 2.14	9.26 ± 2.57	−0.8	=
			CE anteroposterior SI	8.97 ± 1.88	8.86 ± 2.40	9.37 ± 2.25	8.75 ± 2.93	−0.5	=
			CE anteroposterior mean deflection	4.12 ± 2.41	3.74 ± 2.28	4.25 ± 2.36	5.01 ± 3.71	1.1	=
			CE mediolateral SI	7.37 ± 2.30	6.25 ± 2.05	7.47 ± 1.28	5.56 ± 1.38	−0.8	↑
			CE mediolateral mean deflection	2.55 ± 1.49	2.01 ± 1.33	2.75 ± 1.31	2.01 ± 1.34	−0.2	=
	[33]	FIQ	Functional capacity	56.66 ± 11.58	49.81 ± 14.87	48.89 ± 12.08	43.79 ± 12.31	1.7	Δ^#^
		SF-36	Quality of life	33.58 ± 12.10	42.51 ± 11.30	44.16 ± 18.88	54.00 ± 15.83	0.9	Δ↑^#^
		T-Force System	Maximum power of knee extensor muscles	80.92 ± 24.17	85.01 ± 19.73	81.29 ± 28.34	87.05 ± 19.72	1.7	=
			Number of repetitions	22.08 ± 9.21	24.71 ± 6.26	23.75 ± 7.87	28.71 ± 5.25	2.3	Δ
			Muscular fatigue index	0.91 ± 0.14	0.96 ± 0.05	0.90 ± 0.06	0.97 ± 0.17	0.0	=

Sañudo et al.	[28]	Biodex Stability System	OE overall SI	5.53 (1.49)	6.10 (1.40)	7.02 (3.66)	5.75 (2.51)	−1.84	=
			OE mean deflection	4.58 (1.32)	4.99 (1.16)	5.85 (3.18)	4.67 (2.09)	−1.59	=
			OE anteroposterior SI	4.46 (1.36)	5.03 (1.21)	5.36 (2.90)	4.92 (2.18)	−1.01	=
			OE anteroposterior mean deflection	2.18 (1.67)	2.40 (1.08)	2.47 (2.24)	2.48 (1.29)	−0.21	=
			OE mediolateral SI	3.35 (1.17)	3.83 (1.37)	4.54 (2.56)	2.94 (1.44)	−2.08	Δ↑
			OE mediolateral mean deflection	1.23 (1.66)	0.76 (0.19)	2.28 (1.08)	1.31 (0.21)	−0.5	↑
			CE overall SI	9.31 (1.83)	8.89 (1.95)	9.91 (3.64)	9.10 (2.99)	−0.39	=
			CE mean deflection	7.90 (1.77)	7.63 (1.80)	8.43 (3.55)	7.76 (2.83)	−0.4	=
			CE anteroposterior SI	7.31 (1.93)	7.30 (1.84)	7.68 (2.58)	7.32 (2.47)	−0.37	=
			CE anteroposterior mean deflection	3.21 (2.66)	3.87 (2.38)	1.90 (2.01)	3.18 (2.27)	0.62	=
			CE mediolateral SI	5.81 (1.49)	5.21 (1.21)	6.39 (2.86)	5.48 (2.03)	−0.31	Δ
			CE mediolateral mean deflection	1.62 (1.02)	0.95 (0.84)	1.09 (0.87)	1.80 (1.02)	1.38	=
		T-Force System	Number of repetitions	23.72 (7.98)	23.50 (6.63)	30.85 (8.90)	31.14 (7.18)	0.51	=

CG: control group; EG: exercise group; NR: not reported; ELISA: enzyme-linked immunosorbent assay; IGF-1: serum insulin-like growth factor-1; FIQ: fibromyalgia impact questionnaire; SB: static balance; VAS: visual analogue scale; OE: open eyes; CE: closed eyes; SI: stability index; =: no significant difference relative to baseline and/or the control group; **↑**: statistically significant improvement in the WBV group relative to the control group; Δ: statistically significant improvement in the WBV group relative to baseline; #: statistically significant improvement in the control group relative to baseline.

∗Intention-to-treat analysis.

## Appendix

### Levels of Evidence and Conclusion according to the Dutch CBO Guidelines

*Levels of Evidence according to the Dutch CBO Guidelines*

A1: systematic review containing at least two independent trials of level A2;
A2: randomised comparative double-blind study of good quality and sufficient size;
B: comparative trials, but not all characteristics of A2 (also, patient control studies and cohort studies);
C: noncomparative trials;
D: expert opinion.

*Level of Conclusion according to the Dutch CBO Guidelines*. Conclusion is based on the following:

1. research on level A1 of at least two independent trials of level A2;
2. one trial of level A2 or at least two independent trials of level B;
3. one trial of level B or C;
4. expert opinion.

CBO: Institute for Healthcare Improvement.
